# Geometric morphology and population genomics provide insights into the adaptive evolution of *Apis cerana* in Changbai Mountain

**DOI:** 10.1186/s12864-022-08298-x

**Published:** 2022-01-19

**Authors:** Liu Nannan, Liu Huamiao, Ju Yan, Li Xingan, Li Yang, Wang Tianjiao, He Jinming, Niu Qingsheng, Xing Xiumei

**Affiliations:** 1 State Key Laboratory for Molecular Biology of Special Economic Animals, Institute of Special Animal and Plant Sciences of Chinese Academy of Agricultural Sciences, Changchun, People’s Republic of China; 2 Key Laboratory for Bee Genetics and Breeding of Jilin Province, Apiculture Science Institute of Jilin Province, Jilin, People’s Republic of China

**Keywords:** *Apis cerana* in Changbai Mountain, Wing geometry, Population genomics, Genetic differentiation, Genetic diversity, Adaptive evolution

## Abstract

**Background:**

Exploration of adaptive evolution of organisms in response to environmental change can help to understand the evolutionary history of species and the underlying mechanisms of adaptation to local environments, thus guiding future conservation programmes. Before the introduction of *Apis mellifera* in China, eastern honey bees (*Apis cerana*) were the only species used for beekeeping in this region. In the mountains of Changbai, populations of *A. cerana* are considered a distinct ecotype of the species which formed through the distinct selective pressures in this area over time.

**Result:**

We performed a measure of 300 wing specimens of eastern honey bees and obtained the geometric morphological variation in the wing of *A. cerana* in Changbai Mountain. A total of 3,859,573 high-quality SNP loci were yielded via the whole-genome resequencing of 130 individuals in 5 geographic regions.

**Conclusion:**

Corresponding geometric morphology and population genomics confirmed the particularity of the *A. cerana* in Changbai Mountain. Genetic differentiation at the subspecies level exists between populations in Changbai Mountain and remaining geographic regions, and a significant reduction in the effective population size and an excessive degree of inbreeding may be responsible for a substantial loss of population genetic diversity. Candidate genes potentially associated with cold environmental adaptations in populations under natural selection were identified, which may represent local adaptations in populations. Our study provided insights into the evolutionary history and adaptation of *A. cerana* in Changbai Mountain, as well as its conservation.

**Supplementary Information:**

The online version contains supplementary material available at 10.1186/s12864-022-08298-x.

## Background

Nowadays, the number of pollinators has been declining considerably; in particular, the invertebrate pollinators represented by honeybees and butterflies are on the verge of extinction [1, 2]. *Apis cerana* is native to Asia with a long history of managed beekeeping, the populations have also experienced severe declines in recent decades [3, 4].In Northeast China, Korean Peninsula, and even the Russian Far East in a broader sense, populations of *A. cerana* are considered a distinct ecotype of the species [5]. This ecotype wields a crucial role in maintaining the biodiversity of Changbai Mountains. It has adapted to the long winters and short summers of Changbai Mountain. As a crucial part of the ecological food chain in Changbai Mountain, a reduction in the number of existing *A. cerana* bees is bound to affect the survival of upstream predators in the future. In terms of economic value, *A. cerana* in Changbai Mountain bears excellent traits such as rapid reproduction, strong maintenance of colonies, collection capability, resistance to adversity, and cold tolerance [6]. Thus, it is a popular breeding species among beekeepers, and colony breeding and wild hive nectar harvesting remain as an important source of income for people living in economically underdeveloped mountain areas. Unfortunately, the biotic and abiotic factors, such as ecological invasion of *A. mellifera,* habitat loss and fragmentation, overexploitation of biological resources, intensive agricultural development, pathogenic microbial infection, overuse of pesticides, global warming, and atmospheric pollution, have caused a massive decline in the population size and distribution density of *A. cerana* in Changbai Mountain [7, 8]; its population size has decreased from more than 40,000 colonies in 1983 to 19,000 colonies in 2008 [9], and then to less than 10,000 colonies at present, with an alarming decline rate of 75%, resulting in an endangered–sustained state. Understanding the adaptive evolution of organisms towards environmental changes not only helps clarify the evolutionary history of species and the potential mechanism of adapting to the local environment but also guides for future protection plans[10–12]. With the dramatic changes occurring in the genetic structure and diversity of pollinating insects worldwide, considering the changes in climate, ecology, and other environmental factors, introduced or translocated exotic colonies may alter the germplasm gene pool of local *A. cerana* in Changbai Mountain. Collectively, the diversity of *A. cerana* in Changbai Mountain should be further clarified.

Shape and veins of insect wings contain a wealth of ecological and behavioural information [13–15]. Geometric morphology analyses of bee wings have been used for the identification of species and subspecies [16–18]. The wing veins of hymenopteran insects have tended to decrease during the evolutionary history [19]. It has been reported that the capped broods of *Apis cerana cerana* were incubated at constant lower temperature until emergence, resulting in the increase of wing veins of adults, which may be the wing veins that disappeared in the process of evolution [20]. Thus, changes in wing size and morphology under environmental stress can advance our understanding on the adaptive evolution of *A. cerana* in Changbai Mountain. In addition, there are differences in adaptation to various climates between eastern honey bees and western honey bees. It is generally believed that the adaptation of western honey bees to temperate climate conditions is a key step in their evolution [21]. Native bees show higher vitality and environmental tolerance than non-native bees [22–24], therefore *A. cerana* populations inhabiting the mountains of Changbai may have a more effective social defense system than *A. mellifera* through the combination of complex molecular and behavioral mechanisms [25]. *A. cerana* in Changbai Mountain has developed distinct traits in response to mountain conditions, particularly strong resistance to the cold (able to naturally overwinter in extreme cold conditions at − 40 °C) [9]. While population genomics, morphometrics and adaptation of western honey bees to cold environments have been extensively studied [18, 26], a similar assessment on *A. cerana* in Changbai Mountain is lacking.

In this study, we collected samples from 64 sampling sites in the Changbai Mountain, northern, southern, Qinghai–Tibetan, and northwestern regions. Moreover, resequencing data from 66 A*. cerana* samples of each ecotype were downloaded from the literature [27]. A comprehensive analysis of geometric morphometry and population genomics revealed the variation patterns of wing morphology, population genetic diversity, and population history of *A. cerana* in Changbai Mountain, and helped in identifying candidate genes related to their adaptation to the local cold environment. This paper offers an in-depth investigation of the genetic basis and uniqueness of the environmental adaptation of *A. cerana* in Changbai Mountain, thus rendering directions for the scientific and effective conservation of the population.

## Results

### Population genetic structure analysis

**Fig. 1:**
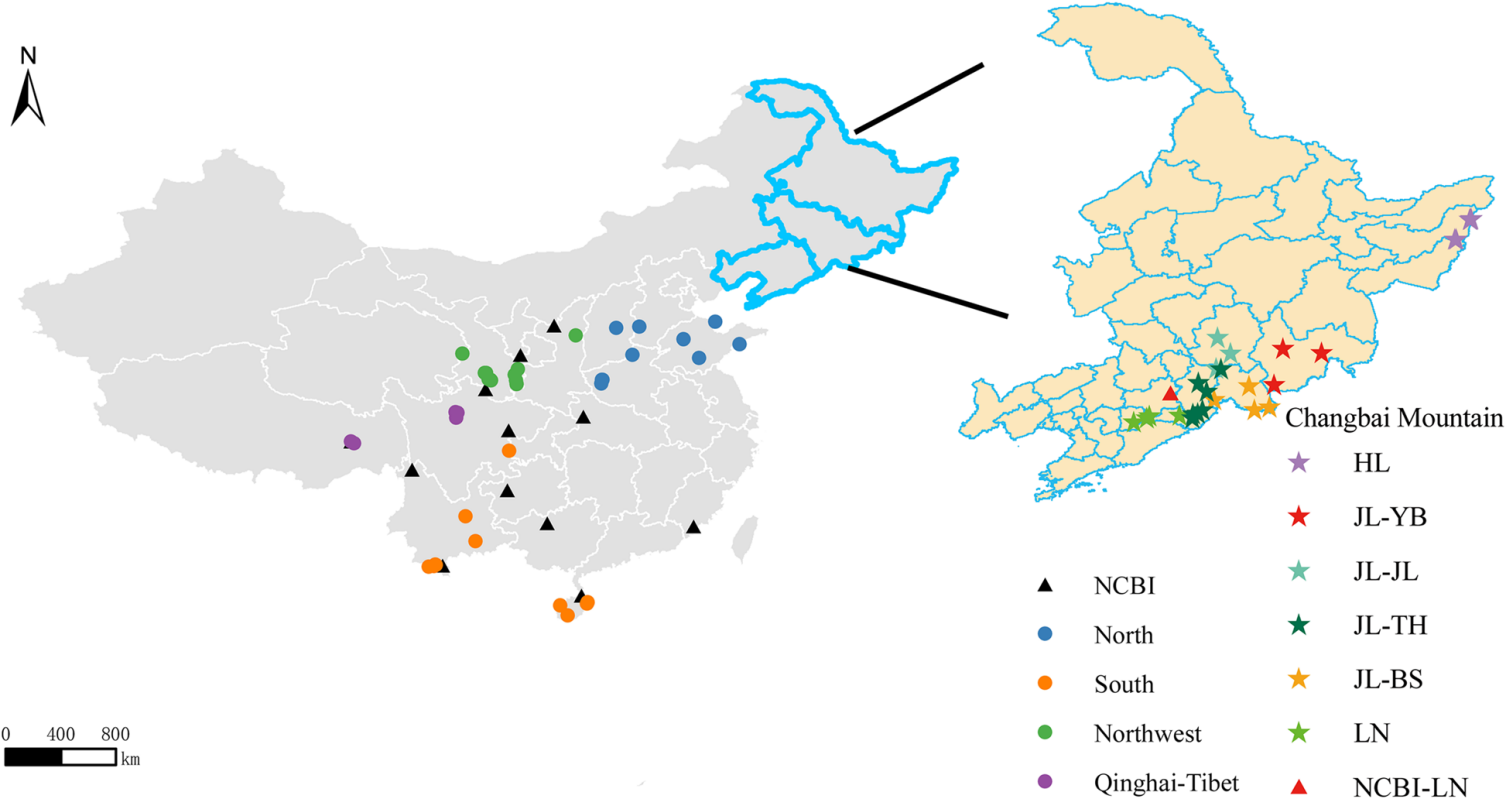
The positions of the 130 individuals in 5 geographic regions in China. The sampling location of the Changbai Mountain area is highlighted as a yellow enlarged area map. Triangles represent the sampling locations of the samples downloaded; circles represent the sampling locations of the samples from northern, southern, northwestern, and Qinghai-Tibetan regions; and stars represent the sampling locations of the samples from Changbai Mountain

**Fig. 2:**
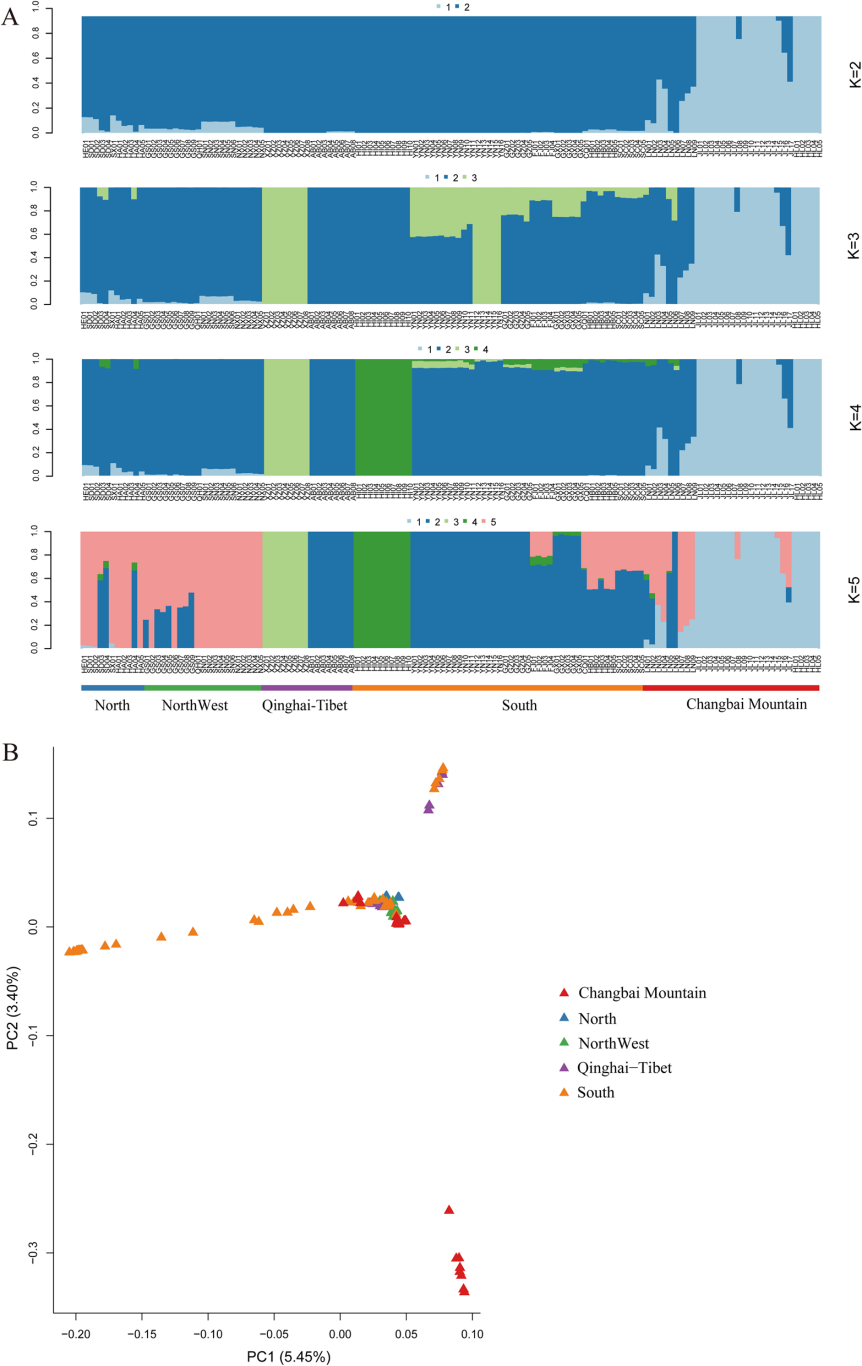
Results of population genetic structure analysis. (**A**) Genetic structure of *A. cerana* populations from 5 regions. Groupings of samples from two to five ancestral clusters are shown. (**B**) Scatter plot of PC1 versus PC2 for the *A. cerana* populations

The samples were collected from North, Northwest, Qinghai-Tibet, South and Changbai Mountain, among which samples from Changbai Mountain were procured covering the entire natural range of the populations (Fig. 1, Additional file 1: Table S1). Whole-genome resequencing yielded a total of 243.37 Gb of data. Genome alignment resulted in an average depth of 13.32 × (Additional file 2: Table S2). For the population genetics analysis, we also downloaded 165.44 Gb of resequenced data of 66 *A. cerana* samples from the published literature (Additional file 3: Table S3). After quality control, a total of 3.86 million SNPs were identified, of which 253,656 were in exonic regions and 52,161 were in non-homologous regions (Additional file 4: Table S4), and a total of 2,237,786 Indels were detected, of which 15,588 were in exonic regions (Additional file 4: Table S5). Population genetic structure was first evaluated using ADMIXTURE [28] (Fig. 2A). The result showed that when *K* = 2–5, most Jilin individuals and all Heilongjiang individuals in Changbai Mountain were from the same ancestor, and all Liaoning individuals were of mixed lineage and had different ancestral origins. In particular, when *K* = 4 and *K* = 5, the origin of the Liaoning individuals was more complex, with individual samples containing a mixture of 3 or more ancestral subgroups, and few Jilin samples showed different degrees of admixture. According to the cross-validation errors, when *K* = 4, the CV error value (0.22113) was the lowest, which was the best *K* value. All regions were divided into 4 genetic components, the individuals from Tibetan (XZ01-XZ08) and Hainan (HI01-HI10) formed an ancestral cluster (Fig. 2A), whereas individuals from Southern region showed a more highly mixed state than the individuals from Northern and Northwestern due to their richness in geographic ecotypes. Afterwards, PCA was performed using GCTA [29] (Fig. 2B), and the results of all three PCAs separated most of the individuals of Changbai Mountain and clustered them together separately (Additional file 5: Fig. S1). The Liaoning individuals (LN01-LN09) were mixed with those from the northern, northwestern, Qinghai–Tibetan, and southern regions, further supporting the results of genetic structure analysis.

### Genetic differentiation and genetic diversity

**Fig. 3:**
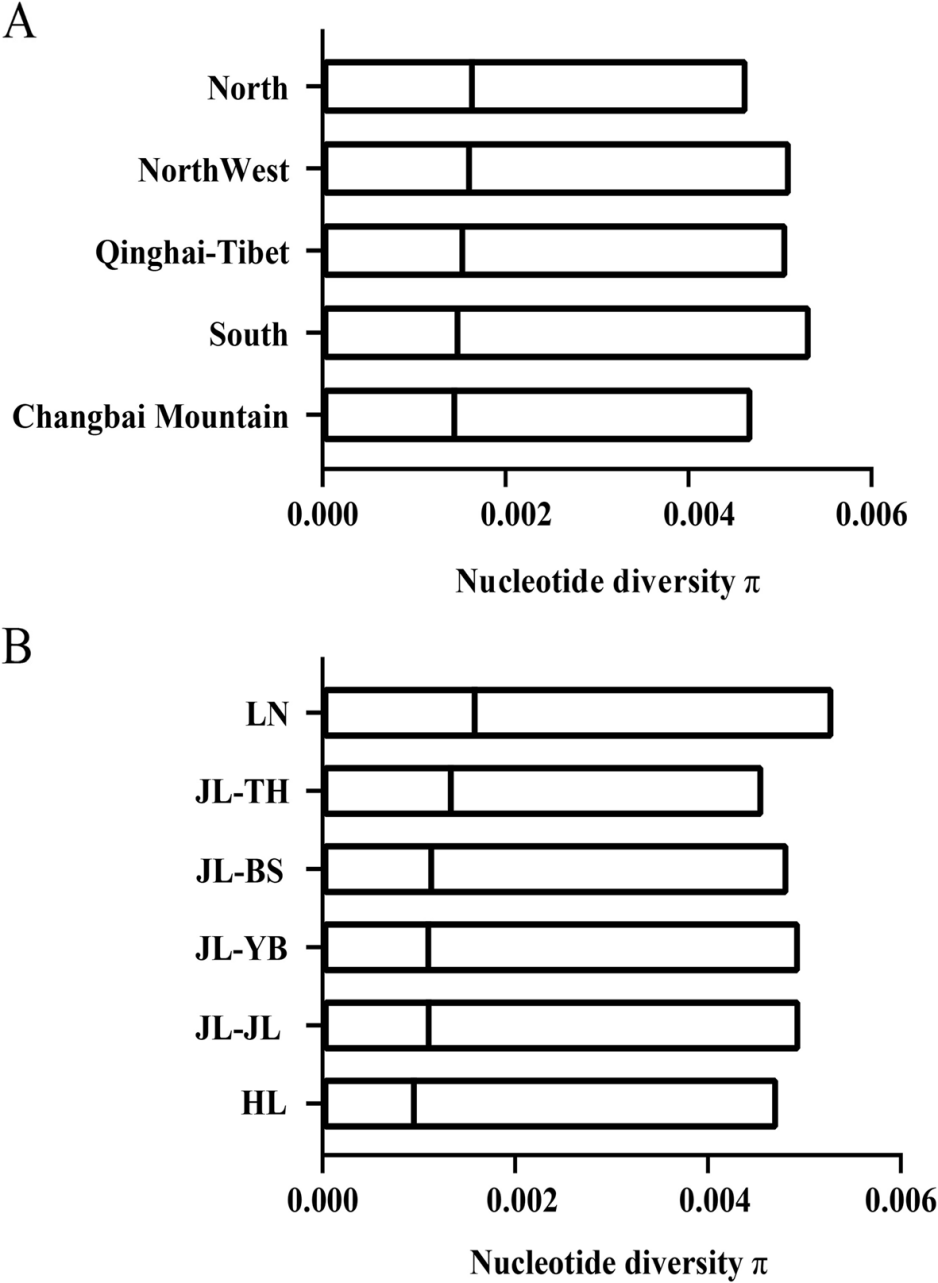
The result of nucleotide diversity(π). (**A**) *A. cerana* from five regions. (**B**) *A. cerana* in Changbai Mountain

The nucleotide diversity (π) and Fst values of *A. cerana* for five geographic regions and Changbai Mountain were analysed. Among *A. cerana* individuals in each geographical region, the highest π was found in the individuals from the North region, and the lowest was in the Changbai Mountain (Fig. 3A). The highest π was found in the Liaoning and the lowest in the Heilongjiang among individuals from Changbai Mountain (Fig. 3B). Paired Fst ranged from 0.0094 to 0.2609, with an average of 0.1133, and *A. cerana* in various regions exhibited moderate genetic differentiation [27, 30, 31]. The mean Fst was 0.2294 between the individuals from Changbai Mountain and the *A. cerana* from the northern, northwestern, southern, and Qinghai–Tibetan regions, showing a high degree of genetic differentiation (Table 1). Paired Fst for samples from each region within the Changbai Mountain individuals ranged from − 0.0031 to 0.2030 (Table 2). Fst for Jilin individuals from JL-BS, JL-JL, JL-TH, and JL-YB were all below 0.02, indicating an extremely low degree of genetic differentiation. The average Fst between the individuals from Heilongjiang and Jilin was 0.1002, which indicated moderate to low differentiation. Notably, the individuals from Liaoning reached a high degree of genetic differentiation, with an average Fst of 0.1506.

**Table 1:** Paired Fst genetic distances between individuals from 5 regions of *A. cerana*

	Changbai Mountain	North	Northwest	Qinhain Tibet
Changbai Mountain	-			
North	0.2214	-		
Northwest	0.2129	0.0094	-	
Quinghai-Tibet	0.2609	0.0534	0.0557	-
South	0.2225	0.0237	0.0282	0.0451

**Table 2:** Paired Fst genetic distances between individuals from Changbai Mountain region

	JL-BS	JL-JL	JL-TH	JL-YB	HL
JL-BS	-				
JL-JL	0.0118	-			
JL-TH	0.0061	-0.0031	-		
JL-YB	0.0088	0.0012	-0.0011	-	
HL	0.1109	0.1111	0.0857	0.0932	-
LN	0.1523	0.1500	0.1027	0.1448	0.2030

**Fig. 4:**
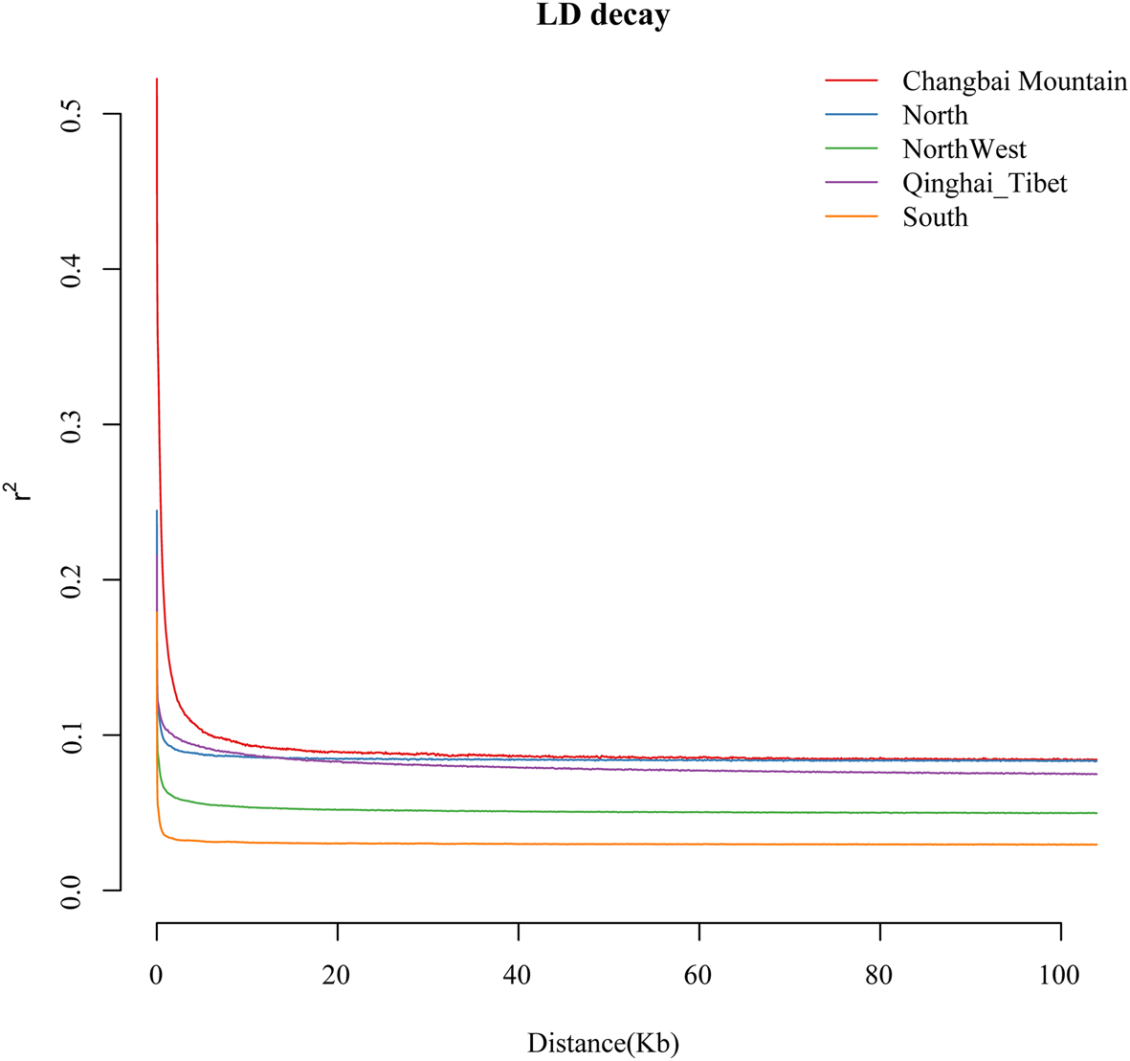
The analysis of LD for *A. cerana*

We evaluated expected heterozygosity (He), observed heterozygosity (Ho), inbreeding coefficient (F), and linkage disequilibrium (LD) for each geographic regions of *A. cerana*, and *A. cerana* in Changbai Mountain had the lowest He, highest Ho, and highest F (Table 3), indicating that they had a low level of genetic diversity, a high level of genetic differentiation from other geographic regions of *A. cerana*, and a high degree of inbreeding. LD analyses showed that the Changbai Mountain region exhibited the slowest LD decay (Fig. 4). Presumably, inbreeding decline may be responsible for the lowest genetic diversity, which is consistent with the results of the analysis of genetic diversity indicators.

**Table 3:** Genetic diversity information of individuals from 5 regions of *A. cerana*

	He	Ho	F
Changbai Mountain	0.1049	1,5227	0.3833
North	0.1638	1,4643	0.0373
Northwest	0.1619	1,4506	0.0385
Qinghai-Tibet	0.1405	1,4609	0.1589
South	0.1504	1,4490	0.0989

### Demographic history

**Fig. 5:**
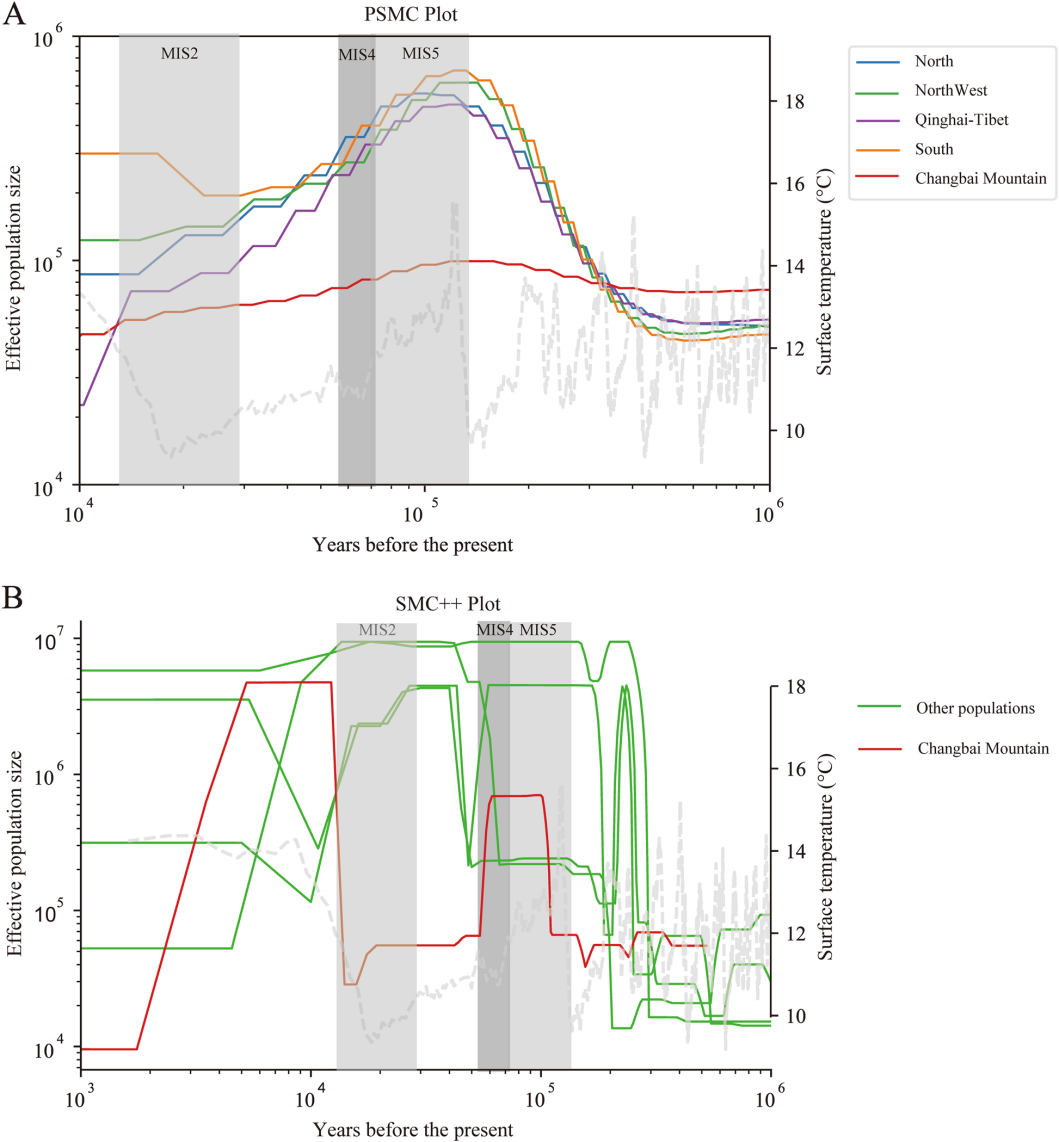
Effective population size of *A. cerana*. Red line represents the effective population size of an *A. cerana* in Changbai Mountain. (**A**) PSMC; (**B**) SMC +  +

The PSMC model is a reliable method to estimate the changes in effective population size [32]. The quantitative variation in the effective population size of *A. cerana* in various regions, including Changbai Mountain populations, coincided with the cyclical variation in historical global temperature, which peaked during the most recent major interglacial period of the Pleistocene, approximately 13,000–11,000 years ago, when the global climate was warm, followed by a sharp decline, consistent with the findings of Chen et al. [27] who reported that the effective population size of *A. cerana* peaked at MIS5. The difference lied in the fact that the *A. cerana* population in Changbai Mountain exhibited a more moderate expansion and contraction of the effective population and the population size was at a lower level than *A. cerana* populations in other regions (Fig. 5A).

The SMC +  + method is an extension of the PSMC method for estimating more precisely the effective population size within 10,000 years [33]. From the MIS4 to the Holocene period, the *A. cerana* population in Changbai Mountain experienced two significant population contractions and one significant population expansion compared with populations in other regions (Fig. 5B).

### Selective sweep analysis

**Fig. 6:**
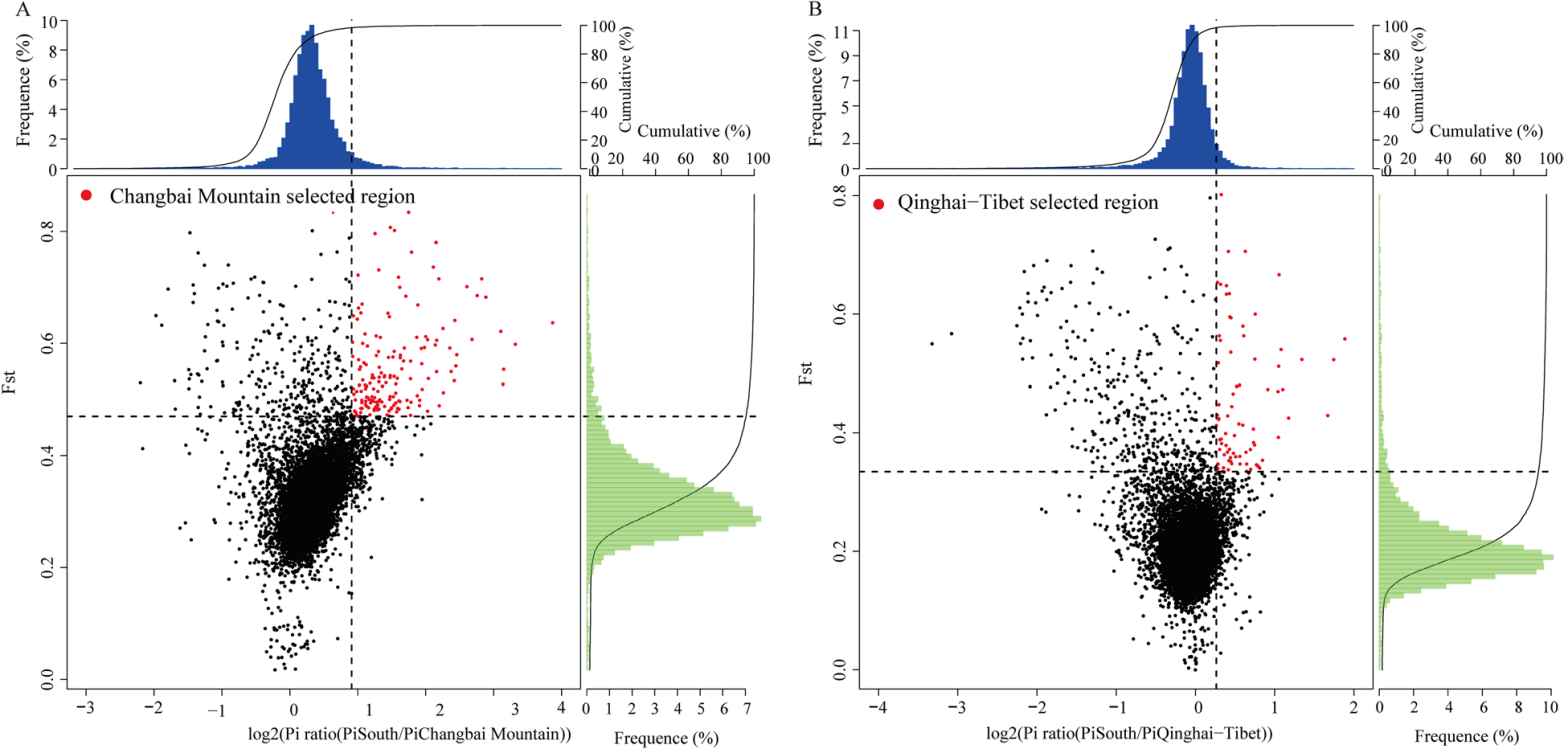
(**A**) Plot of the selective sweep analysis between the individuals from Changbai Mountain and the individuals from Hainan based on Fst and θπ. The horizontal coordinates are the θπ ratio values and the vertical coordinates are the Fst values, which correspond to the frequency distribution plot above and the frequency distribution plot on the right, respectively, and the dot plots in the middle represent the corresponding Fst and θπ ratio values in different windows, where the blue and green areas are the top 5% areas selected by θπ and the red areas are the top 5% areas selected by Fst; (**B**) A selection elimination analysis plot based on Fst and θπ for individuals from Changbai Mountain and Tibet

We performed a selective sweep analysis between Changbai Mountain individuals and Hainan individuals (Additional file 2: Table S2&Additional file 3: Table S3). Genomic regions with the top 5% values for the Fst and θπ ratios (log2(θπ ratio)) in each pairwise comparison were considered to be potentially selective-sweep regions (Fig. 6A). Genes were categorised according to the reference genome for functional gene annotation and KEGG and GO functional categories. A total of 273 genes were selected in the Changbai Mountain population (Additional file 6: Table S6), and they were involved in 1621 GO and 40 KEGG pathways, with 99 GO and 5 KEGG pathways significantly enriched at a threshold of *P* ≤ 0.05 (Additional file 7: Table S7; Table S8).

In this work, we paid considerable attention to the genes related to cold adaptation in the Changbai Mountain region. According to the decreasing order of Fst values, we focused on the genes involved in the top 20 bins. Among these, four structural constituent of cuticle protein and four lipid storage droplets surface-binding protein were included (Additional file 8: Table S9). Based on previous studies on cold tolerance of honeybees, we found many of these genes involved in the GO terms and KEGG pathways to be associated with cold adaptation in insects [34, 35]. In the GO classification, the selected signals were mainly enriched in the metabolism of energy substances, synthesis and production processes, transmembrane transporter activity, ion transport, channel activity, homeostasis, localisation, regulation, binding, enzyme activity, response, signal transduction, and sensory perception. Ether lipid metabolism, glycerophospholipid metabolism, and sphingolipid metabolism are the KEGG pathways in which higher Fst values are jointly involved by selective genes.

The pathway enrichment of the selected genes combined with the functional annotation of the genes themselves resulted in the identification of several highly enriched functional categories, including structural constituent of cuticle (4), zinc ion binding (4), serine/threonine-protein kinase (5), lipid storage droplets surface-binding protein (4), and G-protein-coupled receptor 1 family (4) (Additional file 6: Table S6). Through the selective sweep analysis of population evolution, we attempted to determine the candidate genes that may be related to local adaptation. Because the Tibetan and Changbai Mountain populations belong to the cold-tolerant honeybee ecotype, the same method was used to perform the selective sweep analysis between the Tibetan and Hainan individuals (Fig. 6B). In total, 163 selected genes were obtained for the Tibetan individuals, and 22 genes were identical to the selected region of the Changbai Mountain individuals (Additional file 9: Table S10), of which two enriched genes belonged to the G-protein-coupled receptor 1 family.We speculated that this gene family might wield a role in the genetic basis of cold adaptation in the Changbai Mountain population.

### Morphometric analyses of wings across populations

For the morphological analysis, 30 out of 64 individuals were selected. They were obtained from 6 northern regions, 6 northwestern regions, 3 Qinghai–Tibetan regions, 8 southern regions, and 7 Changbai Mountain regions. Among these, the samples from Changbai Mountain were JL01 (belongs to JL-YB), JL06 (belongs to JL-JL), JL07 (belongs to JL-TH), and JL13 (belongs to JL-BS) in Jilin Province; LN01 and LN09 in Liaoning Province; and HL03 in Heilongjiang Province (Fig. 1; Additional file 1: Table S1).

**Fig. 7:**
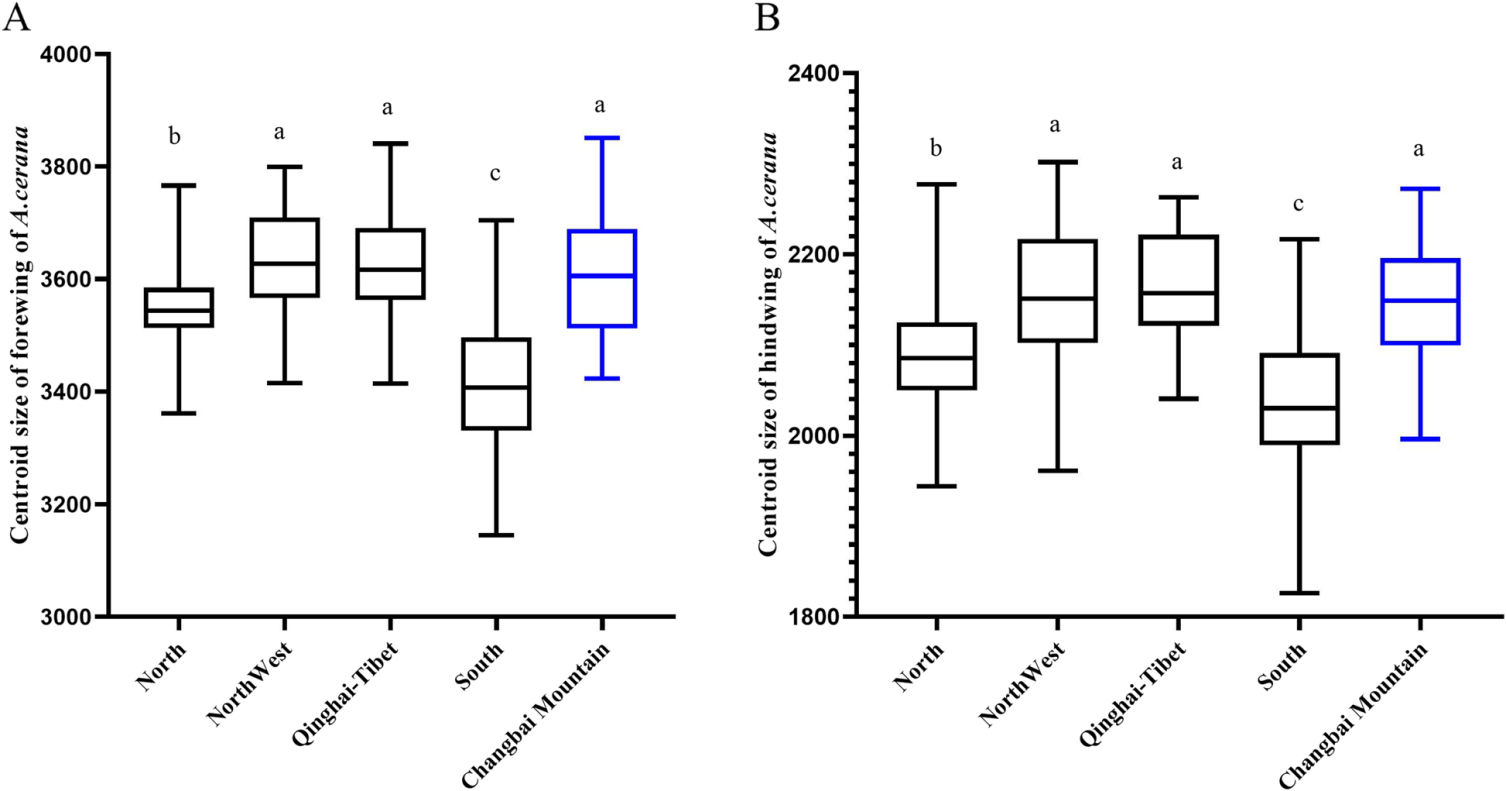
Boxplot of CS of A*. cerana* in wing size across geographical populations. (**A**) Centroid size of forewing of *A. cerana*. (**B**) Centroid size of hindwing of *A. cerana.* Note:Data with different letters indicated significant variations among individuals from each regions at 0.05 level

The wing size is expressed in terms of the centriod size (CS) of the wing. One-way ANOVA revealed a significant difference in CS values of forewings of *A. cerana* distributed in the five geographic regions (*F* = 54.38, *P* < 0.001). The results of multiple tests by the LSD method showed that the individuals in the northwestern region, Qinghai–Tibetan region, and Changbai Mountain had larger forewings, and no notable differences were observed between the individuals. Individuals from Changbai Mountain differed significantly from the individuals in the northern and southern regions in terms of forewing size (*P* < 0.05) (Fig. 7A). Hindwing data demonstrated a highly significant difference in the hindwing CS values among individuals from each region (*F* = 36.08, *P* < 0.001). *A. cerana* from the Qinghai–Tibetan region, northwestern regions, and Changbai Mountain had larger hindwings, and non-significant differences were observed between these individuals, whereas the individuals from Changbai Mountain differed notably (*P* < 0.05) from the northern and southern region individuals (Fig. 7B). Stepwise regression analyses indicated that the mean CS values of forewings and hindwings at each sampling site increased with the latitude and altitude (*P* < 0.05), whereas no significant correlation with longitude was observed (*P* > 0.05) (Additional file 10: Table S11).

The first two principal components of forewings and hindwings occupied 77.74% and 83.29% of the total variance, respectively, both of which were able to illustrate main differences in forewings and hindwings among the geographical regions. Results of forewing PCA showed that individuals from Jilin province and Heilongjiang province were concentrated in the positive half-axis of PC1 and PC2, whereas an individual from Liaoning province (LN09) was in the negative half-axis of PC1 and PC2 (Additional file 11: Fig. S2). Results of hindwing PCA showed that individuals from Jilin province and Heilongjiang province were concentrated in the positive half-axis of PC1, whereas an individual from Liaoning province (LN01) was in the negative half-axis of PC1 (Additional file 11: Fig. S2). Taken together, a certain degree of wing morphological differentiation was observed within *A. cerana* in Changbai Mountain, with no significant differentiation observed between the individuals from Jilin and Heilongjiang, and significant differentiation observed between Liaoning individuals.

## Discussion

A certain number of wild bee colonies are present in Changbai Mountain, mostly in the hollow caves of trees, rock caves in steep mountain cliffs, rock crevices, underground caves, and other hidden places. Eastern honey bees have the germplasm characteristic of flight escape, and a certain number of wild colonies are obtained from various reared colonies and are constantly replenished. In recent decades, human capture and the outbreak of sacbrood virus have greatly changed the population dynamics of eastern honey bees in Changbai Mountain [25, 36]. The wild population suffered a devastating loss, resulting in an increase in the proportion of the wild colonies obtained from the managed colonies. A large reduction in the number of wild bees leads to a decrease in the effective population, which may directly lead to a decrease in the genetic diversity of the *Apis cerana* in Changbai Mountain, and a low level of genetic diversity tends to increase inbreeding. The previous analysis of genetic diversity of *A. cerana* in Changbai Mountain, which were based on SSR and mtDNA markers, also reflected the current situation of a single genetic structure and low genetic diversity [37–39]. It has the highest degree of LD, which is likely to have experienced a serious evolutionary bottleneck event, while showing a very low genetic polymorphism and a slow level of decay. Therefore, we believe that the declining effective population size and loss of genetic diversity explained the main intrinsic factors for the endangerment of *A. cerana* in Changbai Mountain.

The results of population history analysis show that biogeography and climate change have seriously affected the Changbai Mountain population, and glaciation and local volcanic events have likely brought about the bottleneck effect of population history. Available evidence demonstrated that the *A. cerana* was affected by cyclical climate changes during the Quaternary ice age [40], and that volcanic eruption activity at Tianchi, Changbai Mountain, continued throughout the Quaternary [41, 42], inevitably impacting the population evolution and distribution patterns of the existing *A. cerana* in Changbai Mountain. According to the Quaternary glacial remains around the Tianchi volcanic cone, the basic geomorphic features of the Tianchi caldera were formed in the MIS4 and were maintained to the present day after basic stereotypes [42], and the fossil glacier scale of the last glacial period was larger in the early (MIS4) than in the late (MIS2) [43], which implied that the paleoclimate of Changbai Mountain was greatly influenced by glaciation at that time. We therefore focused on labelling the changes in the number of effective population size in the MIS4. MIS4 belongs to the early stage of the last glacial period. The glacier development scale is large. The contraction of the effective population size in the cold period indicated that the decline of global temperature may be detrimental to the development of *A. cerana*.

The population decline during the MIS4, which lasted until the last glacial maximum of approximately 15,000 years ago, is presumed to be mainly due to the paleoglaciation, grand development of the ice sheet, low snowline, and small dust flow, indicative of a cold and wet climate [44], and the harsh natural ecological climate brought about a population decline, which corroborated with the analysis results obtained by the PSMC method. A massive explosive eruptive activity occurred at Tianchi volcano during the Tianwenfeng period at 50,600 years ago, which formed the first breakout crater [45], and climate change and ecological degradation caused by strong volcanism might have aggravated the decrease in *A. cerana*. The effective size of the *A. cerana* in Changbai Mountain increased significantly for once from the last glacial maximum of 15,000 years ago to the Holocene, indicating that global warming was beneficial to the Changbai Mountain population. The period of maximum extent of continental icesheet was 18,000 years ago, and since then, glaciers began to retreat. The Holocene warm period in the northeast was the best period for vegetation development, and the enhanced summer monsoon precipitation due to warming led to ecological recovery [46]. Global warming accelerated the rapid expansion of honeybee populations. The effective population size declined significantly again at approximately 5000–2000 years ago. During the middle Holocene period, approximately 8000–2500 years ago, the climate was warm and humid, the temperature at its peak may have been more than 2 °C higher than modern times [47], the ecological conditions were favourable for species reproduction, while the effective population of honeybees drastically reduced. According to volcanic geology and historical records, there have been several eruptions of the Changbai Mountain 5000 years ago [48, 49], which produced ash drifts up to thousands of kilometers away and deposition thicknesses of several centimeters [50, 51], causing large-scale biological extinctions and serious impacts on the global climate, environment, and ecology [52–54]. In addition, a small peak in the number of hardy plants such as pine, spruce, fir, and birch occurred in the Changbai Mountain around 5000 years ago, which may indicate a climatic cooling event [55], and a similar European Ulmus decline occurred in the pollen fossil belt 5000 years ago in eastern China [56]. Therefore, it is reasonable to speculate that the combination of volcanic heat and abnormal cooling led to a significant decline in the effective population size of *A. cerana* in Changbai Mountain during 5000 years ago.

We explored the selected genes in the evolution of the Changbai Mountain population from multiple perspectives by conducting the selective sweep analysis. The KEGG pathways involved in the selected genes with a high FST value are all related to the metabolism of lipid energy substances, and energy metabolism is crucial for the cold resistance of honeybees [26, 35]. The overwintering period of the Changbai Mountain population lasts for six months, which is a longer period of cold domestication than the colonies living in the warm environment experience in the south, and its cold tolerance is exercised for a long time to develop superior resistance to cold. Cold tolerance exercise in insects is normally an energy-consuming process, and cold tolerance adaptive traits are genetically and evolutionarily stable [57], indicating that the population has evolved a complete adaptive capacity for storage and metabolism of cold-resistant substances under long-term low temperature stress. The result of wing geometric morphometric showed that the CS values of *A. cerana* populations in each geographical region increased with latitude and altitude. Body size in insects may be related to cold resistance, highland populations of *A. cerana* are larger, darker and have longer body hair [12]. The superiority of the Changbai Mountain population for wing and body sizes plays a role in the survival competition of *A. cerana*. Larger wings imply larger insects who adapt to cold conditions at high altitudes and long overwintering periods through an increase in body size [26, 61]. The cold environment of Changbai Mountain challenges the thermoregulation, flight, and reproductive abilities of honeybees, which have larger CS for its forewings and hindwings, implying that individuals can carry more weight in flight [62], have a greater foraging range [63], and have an increased nectar collection ability [64, 65].

**Fig. 8:**
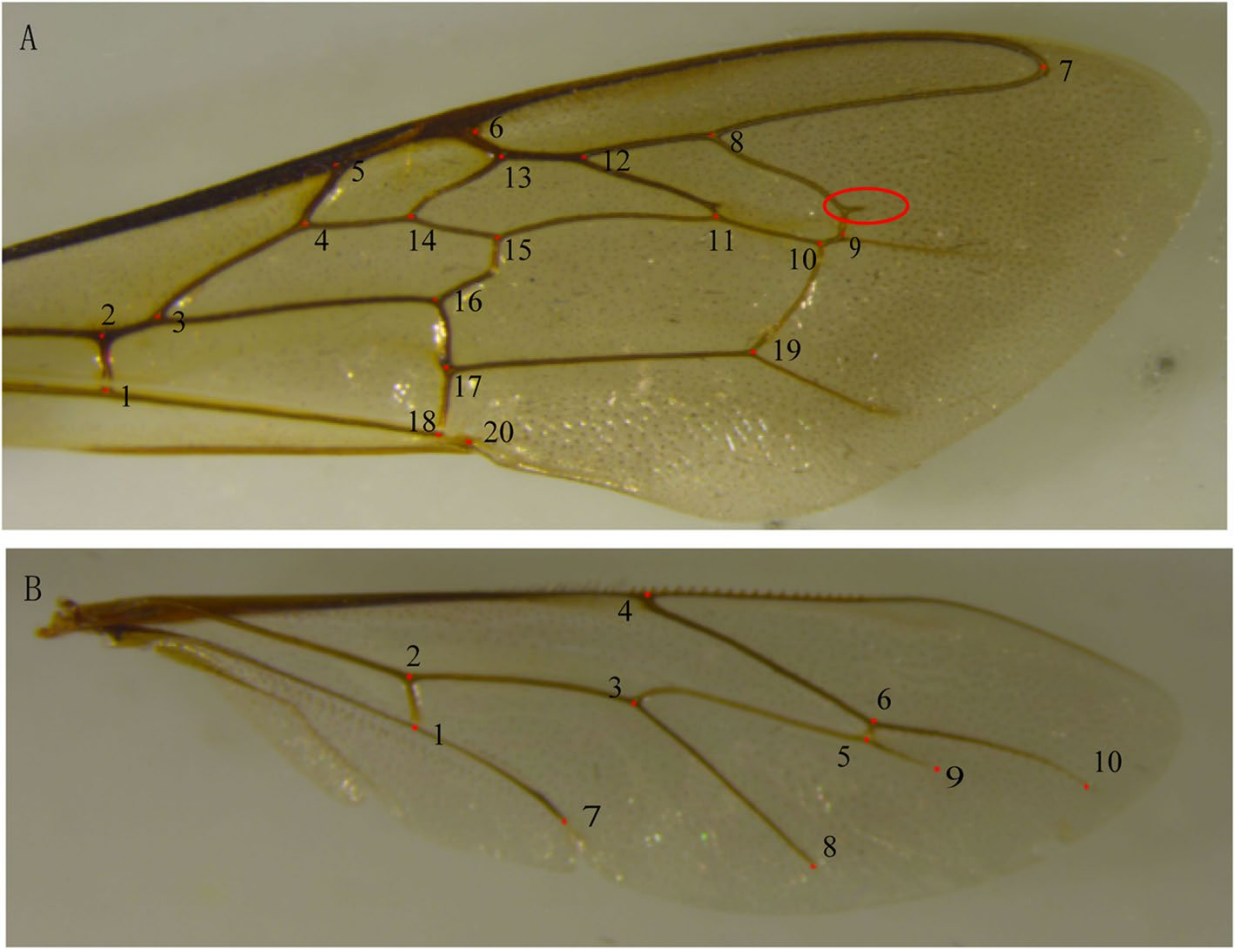
Location of the geometric landmarks on the wing of *A. cerana* in Changbai Mountain. (A) 20 landmarks on the right forewing; the red circle is the small protrusion vein on the radiomedial crossvein. (B) 10 landmarks on the right hindwing

The subspecies-level genetic differences among the individuals from Changbai Mountain and Northern, Northwestern, Southern, and Qinghai-Tibetan regions were observed [30, 31, 66], reflecting the special status of populations in the evolutionary process. Some researchers have suggested that *A. cerana* in Changbai Mountain were morphologically similar to the Korean and Japanese populations, and may be classified as the same taxon [67, 68]. Worker bees usually have a bifurcated protrusion on the radiomedial crossvein and a high cubital index of the forewing, which is a major feature that distinguishes the Changbai Mountain population from other ecotype populations (Fig. 8), and this feature is often used as a bee morphological identification index for genetic classification studies [9]. Zhou et al. [69] confirmed significant morphological differences and similar degrees of differentiation in samples collected from Jilin, Hainan, and Shaanxi, with the cubital index of Jilin *A. cerana* being significantly different from that of the other two honeybees, and suggested that the Jilin population may be a novel subspecies. Gene flow blocking is responsible for population differentiation, prompting differentiation into independent populations that produce genetic traits adapted to the local environment [37]. A recent study revealed the population structure and rapid radiation history of eastern honey bees in mainland Asia, where northeastern populations radiated from a central ancestral group with 6 peripheral subspecies, including Taiwan, Hainan, Bomi, Aba, and Chinghai, and adapted independently to different habitats [70]. On the other hand, *A. cerana* in Changbai Mountain bears varying degrees of genetic differentiation within its population. The genetic differences were negligible among the local individuals of Jilin province, which exhibited a low genetic differentiation compared with Heilongjiang individuals, whereas a significant and major genetic differentiation was noted between the Jilin and Liaoning individuals. It is also proved by the results of wing geometric morphology.

In summary, our research shows that eastern honey bees from Changbai Mountain have genetic and morphological singularity. However, populations in the core area of Changbai Mountain are under threat from the outer reserves, it may lead to irreversible loss of local adaptations in native populations, genetic homogenisation, and pose a risk of genetic contamination [71, 72]. Therefore, local subspecies must be effectively protected. First, it is imperative to strengthen the management of core germplasm reserves and restrain introductions of colonies from non-germplasm core areas to reduce the risk of genetic infiltration of native bees and introduction of pathogens and/or parasites. Second, the feasibility of controlling queen mating should be considered, special areas in the core area where local bees can gather and where mating can be controlled should be designated. Third, conducting basic research on population reproduction, foraging, mating behaviour, and production traits is necessary for successful population conservation, and using appropriate molecular biology techniques to assess the native honeybees population size and regular genetic monitoring of populations will help maintain their purity. Finally, wild colonies experience different selection pressures relative to managed colonies, which can provide insights into the process of adaptation to local environmental conditions, and these honeybees can serve as a genetic reserve gene pool for future managed populations.

## Conclusions

The body and wing sizes of *A. cerana* in Changbai Mountain showed a gradual change with altitude and latitude. A certain degree of wing morphological differentiation was observed within *A. cerana* in Changbai Mountain. Genetic differences were observed between Changbai Mountain and other geographic regions at subspecies level, which has a lower effective population size and genetic diversity, and a higher degree of inbreeding. Selected genomic regions associated with adaptation of populations to cold environments were identified, and these genes may represent local adaptations of population, which serve as excellent candidates for further research. Geometric morphological and colony genomic evidence confirmed the special evolutionary status of *A. cerana* in Changbai Mountain, which will contribute to future conservation programmes.

## Methods

### Honeybee collections

A. cerana samples were collected from 64 sampling sites in 5 geographical regions of China, including northern, northwestern, Qinghai–Tibetan, southern regions, and Changbai Mountain. All the samples were taken from germplasm reserves or original rearing colonies, with priority given to samples from wild hives (e.g. tree cave hives) and traditional-type hives (e.g. tree barrel hives). The samples were transported in anhydrous ethanol and stored in an ultra-low temperature refrigerator at − 80 °C.

### Sequencing, quality control, and alignment reference genome

One workerbee was randomly selected from each colony in the sampling site for whole-genome resequencing. First, genomic DNA was extracted and quantified precisely, and DNA samples with a total volume of ≥ 1.5 μg were used for library construction. The inspected DNA samples were randomly fragmented into 350-bp-long fragments through Covaris, followed by high-quality library construction using the TruSeq Library Construction Kit, the library was prepared through various steps such as end repair, polyA tailing, sequencing junction, purification, and PCR amplification. The constructed library was sequenced by Illumina. Qubit3.0 was then used for preliminary quantification, and the insert size of the library was measured by Agilent 2100 after diluting the library to 1 ng/μL. Then, the effective concentration of the library was accurately quantified by Q-PCR. Finally, according to the effective concentration of library and data output requirements, the Illumina platform was used for PE150 sequencing.

Effective high-quality sequencing data were mapped to the reference genome [73] by using BWA [74] software (set parameter mem -t 4 -k 32 -M), and then the potential PCR duplicates were removed by employing SAMtools [75]. The reference genome version used was GCA_002290385.1_ApisCC1.0_genomic.fa with a genome size of 228,791,026 bp.

### Variation detection and annotation

A total of 130 samples were collected for variant detection. SNP detection was performed using SAMtools [75], and high-quality SNPs were filtered and screened by (1) SNPs with a support number above 3, (2) missing ratio < 10%, and (3) minor allele frequency > 1%. The SNP detection results were then functionally annotated using ANNOVAR software[76].

### Population structures

Based on the results of the population structure analysis, the SNP data obtained from the Jilin and Heilongjiang samples were selected to perform genetic diversity index calculation, LD analysis, population history analysis, and selection elimination analysis. GCTA64 software was used for PCA, and the first three principal components were plotted. The distance matrix was calculated using Treebest-1.9.2 software, and based on this data, a phylogenetic tree was constructed using the neighbour-joining method. The bootstrap values of the tree were obtained after up to 1000 calculations.

### Population genetics analyses

VCFtools was used to calculate π, Fst, He, Ho, and F. For calculating π, the parameter was set to “–window-pi 40,000 –window-pi-step 20,000”; and for calculating Fst, the parameter was set to “–fst-window-size 40,000 –fst-window-step 20,000”.

### Linkage disequilibrium analysis

LD analysis was performed using Haploview software. The SNP spacing on the genome was fitted to the linkage disequilibrium coefficient (r^2^), and data with r^2^ greater than 0.1 were used as the decay value of the LD.

### Effective population size

PSMC and SMC +  + methods were used. The parameters were set as -N30 -t15 -r5 -p 4 + 25*2 + 4 + 6, the mutation rate was set as 5.3 × 10^−9^, and the generation time was set as an estimate of 1 year [26].

### Selective sweep

The *A. cerana* samples from Changbai Mountain (JL01-JL07, JL09-JL14 and HL01-HL05), Hainan (HI01-HI10), and Tibet (XZ01-XZ08) were selected for selection sweep analysis (Additional file 2: Table S2&Additional file 3: Table S3). We compared the Changbai Mountain individuals with the Hainan individuals and the Tibet individuals with the Hainan individuals. We calculated the genome-wide distribution of Fst values and *θπ* ratios for each sliding window (in 20-kb windows with 10-kb step size). The Fst values were Z-transformed, and the *θπ* ratios were log_2_-transformed. The putative selection targets were extracted based on being in the top 5% of log-odds ratios for both Fst and *θπ*. The GO enrichment analysis result was compared with the Pfam database to obtain GO terms, and enrichment analysis was performed using goseq. KOBAS was adopted for KEGG enrichment, the species used was *A. mellifera*, and the BlastX parameter was set to -evalue 1e-5 -m8.

### Dissection and collection of morphometric data

10 worker bees were randomly selected from 30 sampling site, and 300 honeybee samples were collected, which basically covered all climatic regions in China (Additional file 1: Table S1). The right forewings and hindwings of the worker bees were removed with forceps, and the wings were fixed by pressing them in the middle by using two slides to make specimens. The specimens were then photographed using a German Leica LEICA-M165FC fluorescence microscopy imaging system. During image acquisition, all specimens were photographed under the same standard and scale.

The forewings and hindwings of the samples were digitally marked using TPS Util and TPS Dig software, respectively [77, 78], with 20 points for the forewing and 10 points for the hindwing (Fig. 8). All the landmarks were the intersection points of the stable wing veins [18, 79]. The obtained data of the wings were input into Coordgen 6.0 [79], with the 1st and 7th coordinate points as the baseline for the forewings and the 2nd and 10th coordinate points as the baseline for the hindwings. The coordinate values of the landmark were overprinted using the Generalized Procrustes Analysis to remove the influence of non-morphological factors, while the average profile information of each population was calculated on the basis of the GLS method [79].

### Geometric morphometric analysis of wings

One-way ANOVA and LSD multiple tests were performed using SPSS software [79], and the results were presented as box plots. The correlation between wing size variation in elevation, longitude, and latitude was determined by calculating the CS of the wing and regressing the geographical information of the sample sites where they were located. The PCA analyses were performed using PAST software [80].

## Supplementary Information

**Additional file 1. **

**Additional file 2.**

**Additional file 3.**

## Data Availability

All sequencing data generated during the current study have been submitted to the NCBI Short Read Archive (SRA) under the BioProject accession number PRJNA730587. All other data analysed in the present study are included in this published article (and its Supplementary Information files).

## Abbreviations

A cerana: Apis cerana
A mellifera: Apis mellifera
JL-BS: Jilin-Baishan
CS: Centroid size
DNA: Deoxyribonucleic acid
F: Inbreeding coefficient
Fst: F-statistics Genetic differences among population
GO: Gene ontology
He: Expected heterozygosity
HL: Heilongjiang
Ho: Observed Heterozygosity
JL-JL: Jilin-Jilin
KEGG: Kyoto Encyclopedia of Genes and Genomes
KOBAS: KEGG Orthology Based Annotation System
LN: Liaoning
LD: Linkage disequilibrium
LM: Landmark
mtDNA: Mitochondrial deoxyribonucleic acid
MIS: Marine isotope stages
PC: Principal component
PCA: Principal component analysis
π: Nucleic acid diversity
PSMC: Pairwise sequentially markovian coalescent
RH: Raohe
SMC + +: Sequentially markovian coalescent
SSR: Simple sequence repeats
JL-TH: Jilin-Tonghua
TPS: Thin plate spline
JL-YB: Jilin-Yanbian
