# Supplementary material for: Geometric morphology and population genomics provide insights into the adaptive evolution of Apis cerana in Changbai Mountain
Source: BMC Genomics. 2022 Jan 19;23:64. doi: 10.1186/s12864-022-08298-x (PMC8772121; doi:10.1186/s12864-022-08298-x)
Supplement: Supplementary file 1 — Additional file 1. [file 12864_2022_8298_MOESM1_ESM.pdf]

A

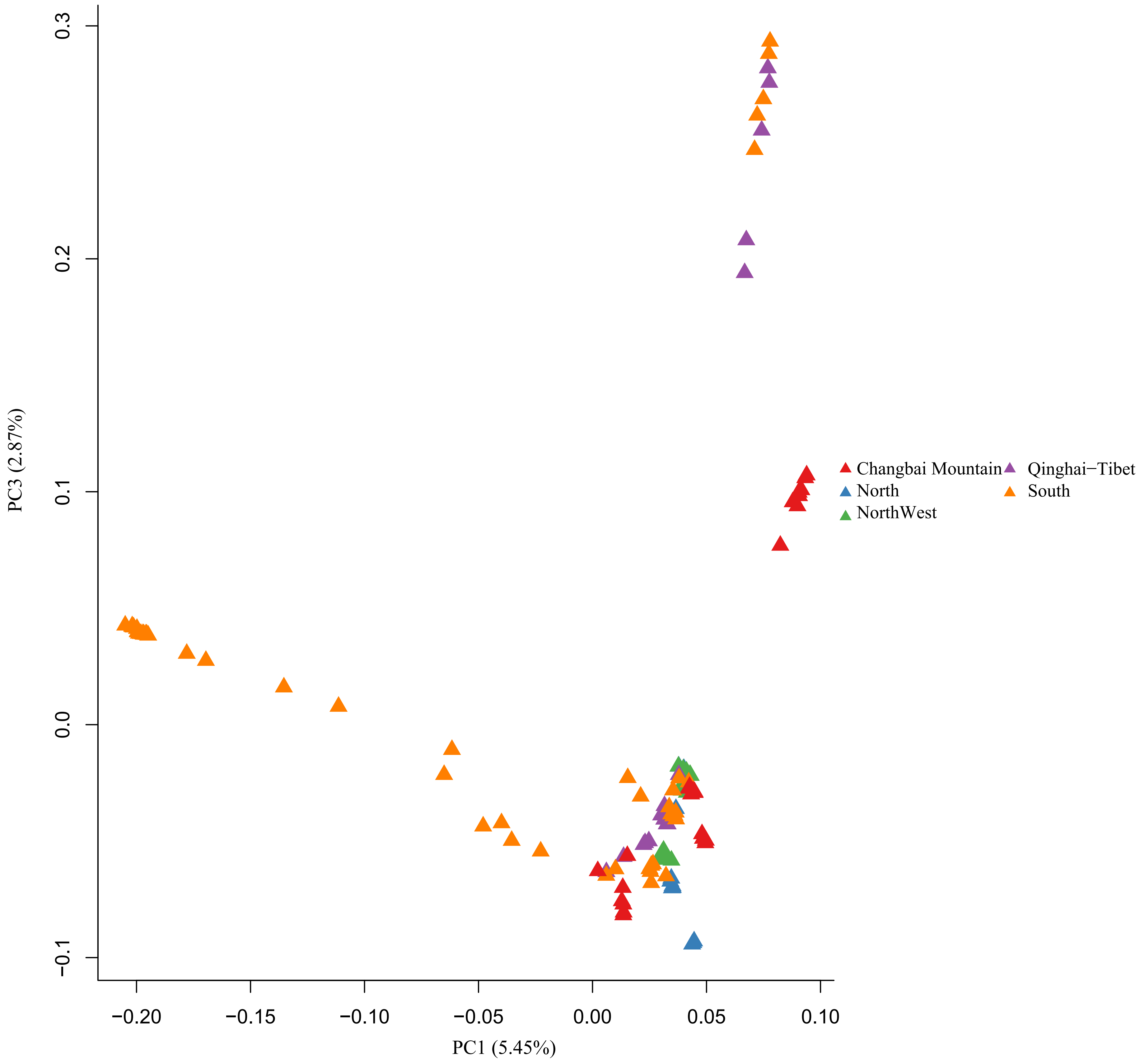

Additional file 5:Fig. S1 The result of PCA. (a) Scatter plot of principal components 1 versus 3 (PC1 vs. PC3) for the *A. cerana.*

B

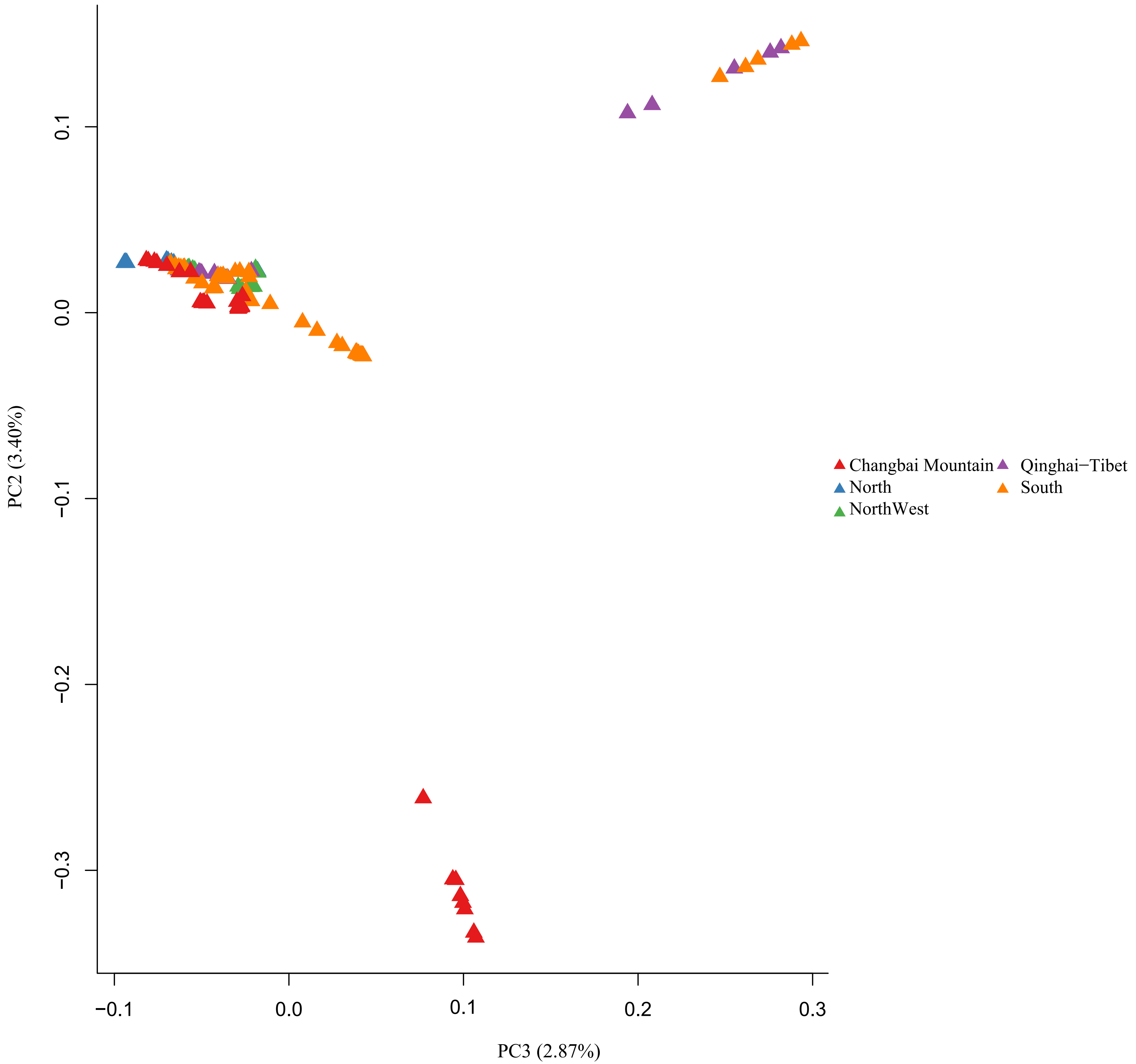

(b) Scatter plot of principal components 2 versus 3 (PC2 vs. PC3) for the *A. cerana*.

A

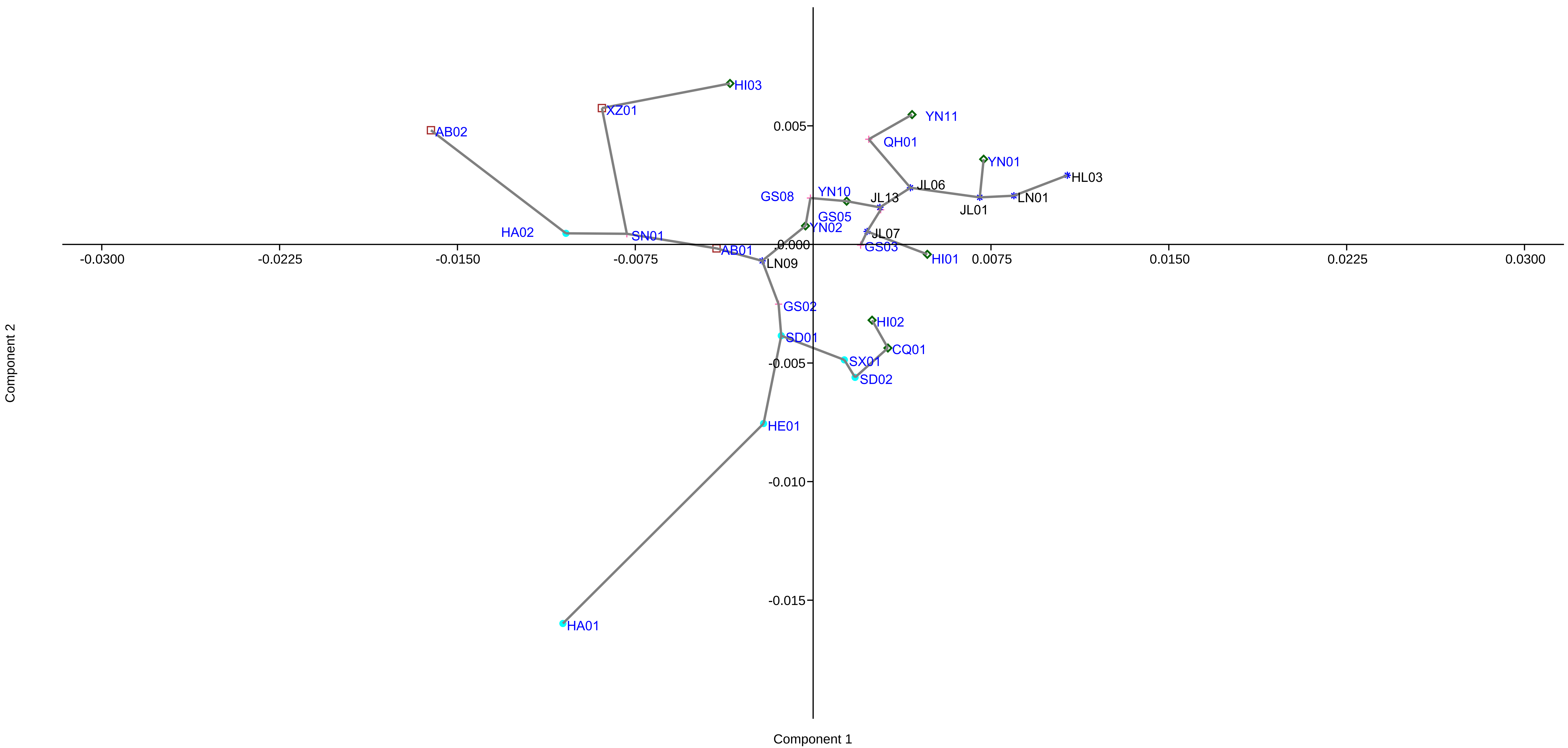

Additional file 11:Fig.S2 The first principal component (Component 1) and the second principal component (Component 2) Min. Spanning tree of the wing of *A.cerana.* (a)The PCA result of forewing;
Note:Dots of different colors represent different geographical dividuals, Changbai Mountain dividuals are marked with blue asterisk(black text).

B

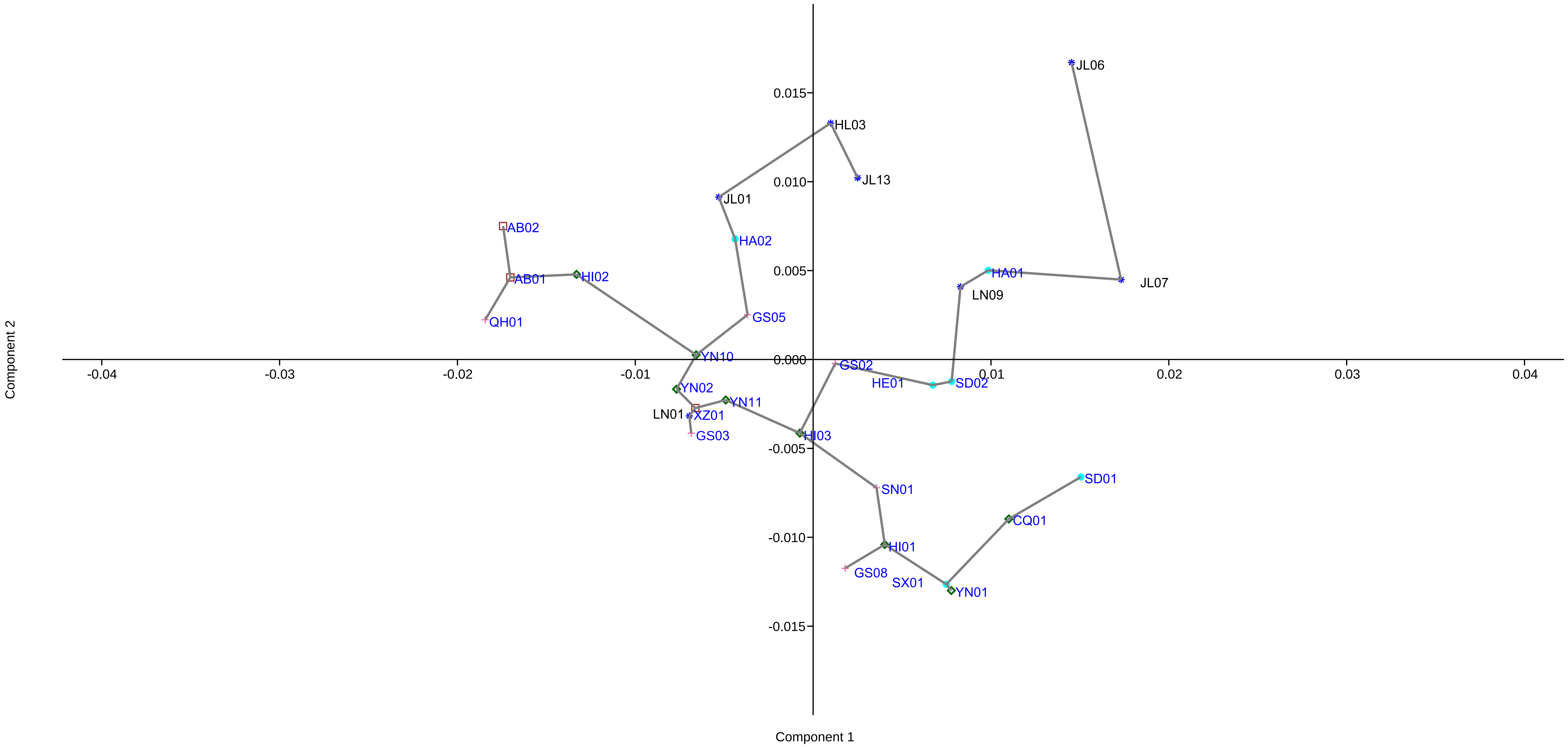

(b)The PCA result of hindwing. Note:Dots of different colors represent different geographical individuals, Changbai Mountain dividuals are marked with blue asterisk(black text).
