# Supplementary material for: Geometric morphology and population genomics provide insights into the adaptive evolution of Apis cerana in Changbai Mountain
Source: BMC Genomics. 2022 Jan 19;23:64. doi: 10.1186/s12864-022-08298-x (PMC8772121; doi:10.1186/s12864-022-08298-x)
Supplement: Supplementary file 2 — Additional file 2. [file 12864_2022_8298_MOESM2_ESM.pdf]

Supplementary Information

Additional file 1: Table S1. Information of 64 sampling sites.

Additional file 2: Table S2. Summary of sequencing data depth, coverage and quality of 64 samples.

Additional file 3: Table S3. Geographic information, sequencing depth and coverage of 66 downloaded samples.

Additional file 4: Table S4. Result of SNP annotation. Table S5. Result of Indel annotation.

Additional file 5: Figure S1. The result of PCA. (a)Scatter plot of principal components 1 versus 3 (PC1 vs. PC3) for the *A. cerana*; (b) Scatter plot of principal components 2 versus 3 (PC2 vs. PC3) for the *A. cerana*.

Additional file 6: Table S6. Selected genes of *A. cerana* in Changbai Mountain.

Additional file 7: Table S7. Enriched GO terms of the selective sweep genes of *A. cerana* in Changbai Mountain ($P < 0.05$). Table S8. KEGG enrichment of the selective sweep genes of *A. cerana* in Changbai Mountain ($P < 0.05$)

Additional file 8: Table S9. Genes in the top 20 selective-sweep regions of Fst value.

Additional file 9: Table S10. Common selected genes of *A. cerana* in Changbai Mountain and Qinghai-Tibet.

Additional file 10: Table S11. The result of the stepwise regression analysis of wing size and geographic information.

Additional file 11: Figure S2. The first principal component (Component 1) and the second principal component (Component 2) Min. Spanning tree of the wing of *A. cerana*. (a)The PCA result of forewing; (b)The PCA result of hindwing.
